# Prognostic value of diabetes and metformin use in a real-life population of head and neck cancer patients

**DOI:** 10.3389/fmed.2023.1252407

**Published:** 2023-09-06

**Authors:** Vincenzo De Falco, Pasquale Vitale, Christian Brancati, Giuseppe Cicero, Annunziata Auriemma, Raffaele Addeo

**Affiliations:** ^1^Medical Oncology Unit, San Giovanni di Dio Hospital, Frattamaggiore, Italy; ^2^Department of Surgical, Oncological and Oral Sciences, Section of Medical Oncology, University of Palermo, Palermo, Italy

**Keywords:** diabetes, head and neck cancer, oral cancer, metformin, HNC, HNSCC

## Abstract

**Introduction:**

Head and neck carcinoma (HNC) is a disease with a poor prognosis despite currently available treatments. The management of patients with this tumor is often complicated by several comorbidities. Among these, diabetes is the second most frequent and its influence on the prognosis is not known.

**Methods:**

In this work, we collected data on progression free survival (PFS) and overall survival (OS) of one hundred twenty-three patients with HNC who received biweekly cetuximab maintenance treatment after first-line chemotherapy. We then compared the survival of nondiabetic patients versus diabetics’ one.

**Results:**

Surprisingly, both PFS (4 vs. 5 months, HR 2.297, *p* < 0.0001) and OS (7 vs. 10 months, HR 3.138, *p* < 0.0001) were in favor of diabetic patients, even after excluding other clinical confounding factors. In addition, we also studied survivals in patients taking metformin, a widely used oral antidiabetic drug that has demonstrated antitumor efficacy in some cancers. Indeed, diabetic patients taking metformin had better PFS and OS than those not taking it, 7 vs. 5 months (HR 0.56, *p* = 0.0187) and 11 vs. 8.5 months (HR 0.53, *p* = 0.017), respectively.

**Discussion:**

In conclusion, real-world outcomes of biweekly cetuximab maintenance remain comparable to clinical trials. The prognostic role of diabetes and metformin was confirmed to be significant in our series, but further prospective studies are needed for a definitive evaluation.

## Introduction

Head and neck cancer (HNC) includes epithelial tumors that originates from oral cavity, pharynx, larynx, paranasal sinuses, nasal cavities, salivary glands. HNC is the seventh most common cancer worldwide with a generally poor prognosis (five-year survival range from 25 to 61% depending on site) (1). Available treatments include chemotherapy, combined or not with radiotherapy, the use of anti-epidermal growth factor receptor (EGFR) drugs such as cetuximab and, recently, also immune checkpoint inhibitors such as pembrolizumab and nivolumab (2). In patients with recurrent or metastatic HNC, prior to the advent of immunotherapy, the addition of cetuximab to first-line platinum (cisplatin or carboplatin) and 5-fluorouracil chemotherapy and the continuation of cetuximab as maintenance therapy in the case of tumor response or disease stabilization significantly improved overall survival (OS), progression-free survival (PFS), and response rate (RR) (3). Maintenance therapy with simplified biweekly (instead of weekly) cetuximab is a safe, effective and feasible alternative therapy in these patients (4, 5).

In cancer patients, comorbidities contribute to determining survival and also determine the oncological treatments that can be administered. This is especially true for HNC patients because they are often elderly patients with other comorbidities with common risk factors such as alcohol and smoking (1). One of the most common comorbidities is type 2 diabetes mellitus (T2DM), which currently affects approximately 500 million people worldwide (6). Already in preclinical models, the consequences of diabetes such as inflammation, hyperglycemia, hyperinsulinemia and increased levels of insulin-like growth factor 1 (IGF-1) have been shown to promote tumor growth (7–9). In particular, the IGF receptor has been shown to activate EGFR and to exert antiapoptotic effects (10–12). The correlation between diabetes and the incidence of some cancers such as liver, pancreas, endometrium, colon and rectum, breast, bladder cancer has been known for many years (7). Instead, the evidence is conflicting on the correlation between diabetes and the risk of developing HNC and on the prognostic value of diabetes in this tumor (13–16). A relationship between the two diseases therefore seems to exist but the mechanisms are complexand it appears to be mediated or confounded by smoking, alcohol use, and body mass index (BMI)/obesity and so requires further elucidation.

In addition, patients with HNC and T2DM undergoing concurrent chemoradiotherapy, compared with patients without diabetes mellitus, experienced higher rates of infection and hematotoxicity, loss of body weight, and higher treatment-related mortality (17).

Several clinical and preclinical studies have demonstrated the efficacy of metformin, an oral antidiabetic, in improving survival and response rate in some types of tumors such as breast (18), colorectal (19), pancreatic (20), esophageal (21), and lung cancer (22). The hypotheses on its anticancer action mainly concern the activation of adenosine monophosphate activated protein kinase (AMPK), which inhibits a pathway involved in the proliferation of cancer cells (23). Furthermore, some mainly retrospective studies have found an impact of metformin also on the survival of patients with HNC (24–26).

In the present retrospective study, we collected survival data in a population with recurrent or metastatic HNC, and compared patients with and without diabetes. Next, in the subgroup of diabetic patients, we analyzed the differences between patients taking metformin and those not taking it.

## Patients and methods

One hundred twenty-three adult patients with recurrent or metastatic HNC were treated with biweekly (q2w) cetuximab in a single institution (Oncology Unit of Hospital “San Giovanni di Dio” in Frattamaggiore, Italy) from December 2016 to May 2019. All patients selected for treatment had histologically verified and evaluable HNC and had received prior platinum-based chemotherapy plus cetuximab, with at least stable disease after the end of this therapy. Patients received cetuximab at 500 mg/m2 over 2 h on day 1 and 15 of each 28 day cycle. Patients continued to receive treatment until disease progression, unacceptable toxicity or patient refusal. Concomitantly adverse events were recorded before each course according to the National Cancer Institute Common Terminology Criteria for Adverse Events (CTCAE) version 3.0. If patients developed grade 3-skin toxicity, the dose of cetuximab was postponed until recovery to grade 2; in those with recurrent episodes of grade 3 skin toxicity, the dose of cetuximab was reduced by 20% in the subsequent treatment cycles. Patients classified as “diabetics” were initially diagnosed according to the American Diabetes Association criteria (casual plasma glucose concentration ≥ 200 mg/dL or fasting plasma glucose ≥126 mg/dL or 2 h glucose ≥200 mg/dL after Oral Glucose Tolerance Test). BMI was calculated through the body mass (kg) divided by the square of the body height (m). It was used to differentiate underweight (<18.5 kg/m^2^), normal (18.5–24.9 kg/m^2^), overweight (25–29.9 kg/m^2^), and obese individuals (>30.0 kg/m^2^) according to World Health Organization (WHO).

Data were collected starting from maintenance treatment with cetuximab. The retrospective study protocol was approved by the board at the study site (ASL Napoli 2 Nord). All patient information was recorded in an internal computer database. The study was performed in accordance with the Declaration of Helsinki and Good Clinical Practice guidelines. All patients signed a written informed consent and agreed with the research use of their anonymized data.

### Statistical analysis

Survival curves were generated based on the Kaplan–Meier method. OS was defined as time from cetuximab initiation date to death of any cause, censored at last follow-up date; PFS was defined as time from cetuximab initiation date to any failure, censored at the last follow-up date. Statistical significance of survival curves was calculated using the Log-rank test. Cox proportional hazards regression models were used for multivariable analysis (MVA). Graph Pad v.9.5 was used to generate survival curves and to calculate statistics throughout the entire manuscript. A *p* value of less than 0.05 was considered statistically significant.

## Results

### Patients’ characteristics

123 patients were enrolled in the analysis. Baseline patient characteristics are summarized in Table 1. The median age was 65 years with a preponderance of males over females (76.4% vs. 23.6%). 48.8% had a BMI in the normal range or higher. 48.8% were smokers and 31.7% had moderate to heavy alcohol consumption. Within the total population, 57 (46.3%) patients had a diagnosis of T2DM, of which 31 (54.4%) were on metformin at baseline. Furthermore, the most frequent sites of HNC were the larynx (41.5%), oral cavity (16.3%), oropharynx (14.6%) and hypopharynx (13.8%). The majority of patients had distant metastatic disease (69.9%) and all had induction chemotherapy with cisplatin (64.2%) or carboplatin (35.8%) plus fluorouracil.

**Table 1:** Patients’ baseline characteristics.

	All population	Patients with type 2 diabetes mellitus (T2DM) taking metformin	Patients with type 2 diabetes mellitus (T2DM) NOT taking metformin
Patients (*n*, %)	123	31 (25.2%)	26 (21.1%)
Median age, years	64	69	64.5
Age ≥ 70 years (*n*, %)	36 (29.3%)	15 (48.4%)	6 (23.1%)
Sex (male/female)	94/29	24/7	20/6
ECOG performance status (*n*, %):			
0	36 (29.3%)	9 (29%)	7 (26.9%)
1	56 (45.5%)	14 (45.6%)	11 (42.3%)
2	31 (25.2%)	8 (25.8%)	8 (30.8%)
HPV positive	6 (4.9%)	2 (6.5%)	1 (3.8%)
Smokers	60 (48.8%)	12 (38.7%)	13 (50%)
Moderate or heavy alcohol consumption	39 (31.7%)	9 (29%)	9 (34.6%)
Body mass index:			
Obese (>30.0 kg/m^2^)	4 (3.3%)	1 (3.2%)	1 (3.8%)
Overweight (≥25 kg/m^2^)	9 (7.3%)	2 (6.5%)	2 (7.7%)
Normal (≥18.5 kg/m^2^ and < 25 kg/m^2^)	47 (38.2%)	11 (35.5%)	10 (38.5%)
Underweight (<18.5 kg/m^2^)	63 (51.2%)	17 (54.8%)	13 (50%)
Subsite:			
Larynx	51(41.4%)	16 (51.6%)	9 (34.6%)
Oral cavity	20 (16.3%)	2 (6.5%)	5 (19.2%)
Oropharynx	18 (14.6%)	5 (16.1%)	6 (23.1%)
Hypopharynx	17 (13.8%)	1 (3.2%)	3 (11.5%)
Paranasal sinus/nasal cavity	6 (4.9%)	3 (9.7%)	0
Nasopharynx	7 (22.6%)	3 (9.7%)	1 (3.8%)
Neck node, unknown primary	4 (3.3%)	1 (3.2%)	2 (7.7%)
Disease stage:			
Locoregional recurrence only	37 (30%)	9 (29%)	8 (30.7%)
Metastatic disease only	61 (49.6%)	15 (48.3%)	12 (46.2%)
Locoregional and metastatic disease	25 (20.3%)	7 (22.6%)	6 (23.1%)
Received prior radiotherapy	123 (100%)	31 (100%)	26 (100%)
Induction chemotherapy for recurrence or metastatic disease:			
Cisplatin plus 5-fluoruracil	79 (64.2%)	20 (64.5%)	17 (65.4%)
Carboplatin plus 5-fluorouracil	44 (35.8%)	11 (35.5%)	9 (34.6%)

### Efficacy and safety of maintenance treatment with cetuximab

**Figure 1:**
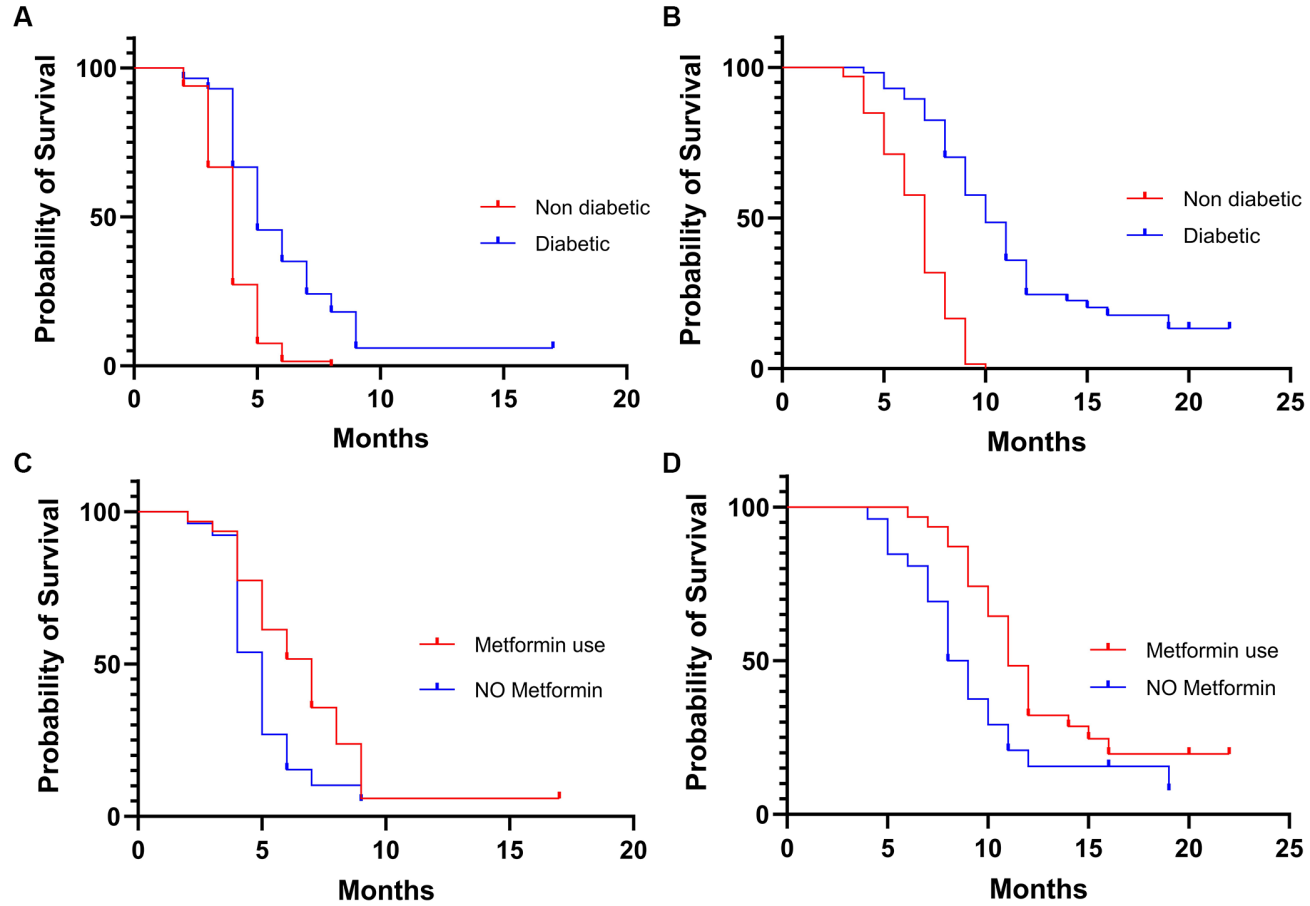
Kaplan–Meier of progression free survival **(A)** and overall survival **(B)** in the overall population. Kaplan–Meier of progression free survival **(C)** and overall survival **(D)** in the diabetic population.

The entire study population started biweekly cetuximab as maintenance treatment after chemotherapy induction and radiotherapy. Median follow-up period was 16 months. Most patients had disease control (58.5%) during cetuximab, while 19.5% had an objective response to treatment (Table 2). In patients with T2DM, the objective response rate was higher in patients taking metformin (29% vs. 19.2%). PFS in the general population was 4 months, while OS was 8 months. No survival differences were observed considering Body Mass Index (BMI) for underweighted versus normal and overweighted individuals (Table 2). Both PFS (Figure 1A) and OS (Figure 1B) disfavored the subgroup of patients without T2DM compared with those with T2DM, respectively 4 vs. 5 months (Hazard Ratio (HR) 2.297, *p* < 0.0001) and 7 vs. 10 months (HR 3.138, *p* < 0.0001). Furthermore, we compared the survival of diabetic patients who took metformin versus those who did not. The metformin group had a survival advantage in terms of both PFS (7 vs. 5 months, HR 0.56, *p* = 0.0187) (Figure 1C) and OS (11 vs. 8.5 months, HR 0.53, *p* = 0.0170) (Figure 1D).

**Table 2:** Efficacy of maintenance treatment with cetuximab.

	Events/number all population of study (*n* = 123)	Patients with type 2 diabetes mellitus (T2DM) taking metformin (*n* = 31)	Patients with type 2 diabetes mellitus (T2DM) NOT taking metformin (*n* = 26)
Response rate:			
Complete response (CR)	2 (1.6%)	1 (3.2%)	1 (3.8%)
Partial response (PR)	22 (17.9%)	8 (25.8%)	4 (15.4%)
Stable disease (SD)	48 (39%)	11 (35.5%)	10 (38.5%)
Disease control (CR + PR + SD)	72 (58.5%)	20 (64.5%)	15 (57.6%)
Progression disease (PD)	50 (40.7%)	11 (35.5%)	11 (42.3%)
Not evaluable	1 (0.8%)	–	–
PFS, months (95% CI)	4.0	7 (95%CI 5–8)	5 (95%CI 5–6)
OS, months (95% CI)	8.0	11.0 (95%CI 10–12)	8.5 (95%CI 7–10)

These data were also confirmed using Cox regression and entering variables such as sex, age, performance status, smoking habits and alcohol habits. Indeed, in the study population, diabetes is a protective factor in patients with HNC, both in terms of PFS and OS (Table 3). Conversely, for PFS, older age and smoking status have a negative effect on prognosis. Smoking, on the other hand, does not have a statistically significant effect. Analyzing the data relating to metformin with Cox regression, it is confirmed as a positive prognostic factor. This is statistically significant in terms of OS while for PFS there is only a trend in favor of its use (Table 3).

**Table 3:** Multivariate analysis for progression free survival (PFS) and overall survival (OS).

		PFS		OS	
Variable	Comparison	HR (95% CI)	*p* value	HR (95% CI)	*p* value
(A) Overall population					
Sex	Female vs. Male	0.9882 (0.6198–1.530)	0.9588	0.9746 (0.6097–1.516)	0.9114
Age	(continuous)	0.9612 (0.9341–0.9890)	0.0066	0.9964 (0.9680–1.026)	0.8071
ECOG status	(continuous)	1.171 (0.8937–1.536)	0.2514	1.187 (0.9080–1.554)	0.2108
Smoke habit	Yes vs. No	1.220 (0.8285–1.798)	0.3136	1.048 (0.7010–1.568)	0.8195
Alcohol consumption	Yes vs. No	1.821 (1.156–2.837)	0.0087	1.110 (0.7223–1.675)	0.6254
Diabetes	Yes vs. No	0.3086 (0.2013–0.4674)	<0.0001	0.1599 (0.0947–0.2618)	<0.0001
(B) Diabetic population					
Sex	Female vs. Male	0.9101 (0.4027–1.911)	0.8110	1.217 (0.5534–2.529)	0.6095
Age	(continuous)	0.9764 (0.9340–1.021)	0.2906	0.9907 (0.9439–1.041)	0.7071
ECOG status	(continuous)	1.410 (0.9429–2.121)	0.0954	1.249 (0.8237–1.922)	0.3013
Smoke habit	Yes vs. No	1.449 (0.7794–2.688)	0.2378	1.223 (0.6289–2.374)	0.5503
Alcohol consumption	Yes vs. No	2.301 (1.079–4.928)	0.0305	1.069 (0.5302–2.083)	0.8473
Metformin use	Yes vs. No	0.5827 (0.3148–1.085)	0.0851	0.5229 (0.2778–0.9870)	0.0438

Adverse events (AEs) during cetuximab treatment are shown in Table 4. Treatment was generally well tolerated with an acceptable rate of adverse events, rarely grade > 2 (according to CTCAE). The most common were fatigue, hypomagnesemia and typical cutaneous adverse events (acneiform rash, desquamation, nail disorders). No differences in toxicity, related to cetuximab treatment, were observed in the diabetic population.

**Table 4:** Adverse events in the overall population and in the type 2 diabetes mellitus (T2DM) subgroup.

Adverse event	Overall population (*n* = 123)		T2DM patients (*n* = 57)	
	Any grade (%)	Grade 3 (%)	Any grade (%)	Grade 3 (%)
Fatigue	69 (56.1)	0	32 (56.1)	1 (1.8)
Acneiform rash	67 (54.5)	3 (2.4)	30 (52.6)	3 (5.3)
Hypomagnesemia	49 (39.8)	1 (0.8)	23 (40.4)	2 (3.5)
Desquamation	31 (25.2)	1 (0.8)	12 (21.1)	1 (1.8)
Mucositis	16 (13)	1 (0.8)	6 (10.5)	0
Nail disorders	13 (10.6)	0	5 (8.8)	0
Pruritis	10 (8.1)	0	4 (7)	0
Nausea	8 (6.5)	0	3 (5.3)	0
Infusion reaction	5 (4.1)	0	2 (3.5)	0
Muscle weakness	5 (4.1)	0	2 (3.5)	0
Diarrhea	5 (4.1)	1 (0.8)	2 (3.5)	0

## Discussion

To date, HNCs still represent tumors with a poor prognosis despite available therapies. Due to the characteristics like location and risk factors (especially smoking and alcohol), this type of tumor often affects patients with other comorbidities. The most frequent non-neoplastic comorbidities in these patients are pulmonary disease (17.9%), diabetes mellitus (7.9%), myocardial infarction (6.7%), and peptic ulcer disease (5.2%) (27).

There are several studies that have demonstrated an increased risk of HNC in patients with diabetes (13, 28–32) and others that have also demonstrated a worse prognosis of these patients compared to non-diabetic patients (15, 29). However, there are some studies on populations with HNC that contradict these data, demonstrating that there is no correlation with diabetes, either as a risk factor (33–35) or as a negative prognostic factor (36, 37). Surprisingly, in our study, patients with diabetes have a better survival, both in terms of PFS and OS. This is confirmed by excluding other confounding factors such as gender, age, performance status, and alcohol and smoking habits. These data, even considering the limitations of the study, could be influenced by two factors. One could relate to the involvement of the insulin growth factor (IGF-1) pathway. In fact, it has been demonstrated that IGF-1 influences tumor growth, having a mitogenic and antiapoptotic role (38, 39). IGF-1 performs its role through its receptors (IGF-1R) and the levels of IGF-1 are influenced by the levels of circulating proteins that bind to it (IGF-BP). Since insulin increases IGF-1 levels and decreases IGF-BP levels, and insulin levels are subnormal in diabetics, this may explain the better prognosis of these patients. Indeed, some works have shown that elevated expression of IGF-BP-3 was associated with a shorter time to progression in HNC patients (40) and that levels of IGF factors were found to be maximum in stages with better prognosis in oral cancer (41). The other factor that could positively influence the prognosis of these patients is the use of metformin. In fact, this drug has been studied in numerous types of cancer for its presumed antitumor effects (41), however the presence of trials with an adequate study design and number, did not lead to a definitive conclusion regarding its efficacy. Just for HNCs, there have also been numerous studies, including meta-analyses (24–26, 42, 43), that have shown a positive prognostic association between metformin use and cancer (44, 45), particularly in patients with oral cancer (46, 47), hypopharyngeal carcinoma (24), laryngeal carcinoma (42, 48). In our study as well, we found a better survival in diabetic patients who took metformin compared to those who took other hypoglycemic drugs, even excluding other confounding factors. In addition, metformin has been shown as a radiosensitizer in colorectal cancer, pancreatic cancer, esophagus cancer (24) but there are few data on HNC (11). However, this may be another factor that influenced the results of our study, as all of the population received radiotherapy for HNC before starting cetuximab. However, there are some studies that have not shown any benefit of metformin in patients with HNC and that should be taken into consideration (26, 49).

Our study has some limitations including the retrospective nature, the sample size, a selection bias due to the single medical center, and the presence of other confounding factors not considered, however it represents an important description of real clinical practice. In conclusion, the correlation between the prognosis of patients with HNC and diabetes, as well as that with metformin use, needs further study. In particular, prospective studies evaluating the use of metformin also in non-diabetic HNC patients represent an important goal in the future of oncology.

## Data availability statement

The raw data supporting the conclusions of this article will be made available by the authors, without undue reservation.

## Ethics statement

The studies involving humans were approved by Comitato etico ASL Napoli 2 Nord. The studies were conducted in accordance with the local legislation and institutional requirements. The participants provided their written informed consent to participate in this study.

## Author contributions

VF, PV, and RA contributed to the conceptualization, investigation, and writing—original draft preparation. VF, PV, CB, GC, AA, and RA contributed to writing—review and editing of the article. All authors contributed to the article and approved the submitted version.

## Conflict of interest

The authors declare that the research was conducted in the absence of any commercial or financial relationships that could be construed as a potential conflict of interest.

## Publisher’s note

All claims expressed in this article are solely those of the authors and do not necessarily represent those of their affiliated organizations, or those of the publisher, the editors and the reviewers. Any product that may be evaluated in this article, or claim that may be made by its manufacturer, is not guaranteed or endorsed by the publisher.
